# Emergency Department Presentations of West Nile Virus

**DOI:** 10.5811/westjem.47475

**Published:** 2025-12-24

**Authors:** Kylie Jenkins, Wayne Martini, Alyssa K. McGary, Heidi E. Kosiorek, Nicole R. Hodgson

**Affiliations:** * Creighton University School of Medicine, Department of Emergency Medicine, Phoenix, Arizona; † Mayo Clinic Arizona, Department of Emergency Medicine, Phoenix, Arizona; ‡ Mayo Clinic, Department of Quantitative Health Sciences, Scottsdale, Arizona

## Abstract

**Introduction:**

Maricopa County, Arizona, experienced its largest West Nile virus outbreak in 2021, with 1,487 cases and 101 deaths, in the midst of the COVID-19 pandemic. We sought to describe initial presentations of emergency department (ED) patients ultimately diagnosed with West Nile virus and determine how often patients presented to the ED before their diagnosis. To assist with disease recognition during future outbreaks, we examined in detail cases where emergency physicians initially did not suspect West Nile virus.

**Methods:**

We reviewed records from May–December 2021 for patients with a positive West Nile virus result and at least one ED visit within 15 days. Data included age, sex, race, Emergency Severity Index (ESI) score, number of ED visits, chief complaint, vital signs, blood or cerebrospinal fluid (CSF) testing, diagnosis, and disposition. We excluded cases with only immoglobulin G-positive results or outpatient tests, leaving 147 cases.

**Results:**

Among 147 ED West Nile virus cases, the median patient age was 67 years, with patients being predominantly male (66.7%) and White (97.3%). The most common presenting chief complaints included fever (23.8%), headache (17.7%), and generalized weakness (11.6%). Emergency physicians initiated testing for the virus in 63 cases (42.9%). Patients dispositioned (n = 84, either discharged or admitted) from the ED without initiation of testing tended to be older (median 73 vs 62 years, *P* < .001), with higher triage respiratory rate (mean 19.4 vs 18.3 breaths per minute, *P* = .05) and lower triage oxygen saturation (median 96% vs 97%; *P* =.02). Emergency physicians predominantly performed CSF testing (n = 42 patients) over serum testing (n = 21 patients). Patients tested via CSF had lower ESI scores than those tested via serum (ESI score of 1–2 45.3% vs 14.3%, *P* = .03).

**Conclusion:**

Emergency physicians did not initiate testing in 57.1% of initial ED encounters of patients ultimately found to have West Nile virus. During West Nile virus outbreaks, emergency physicians should stay vigilant for less acute presentations, such as generalized weakness in elderly patients, along with typical presentations including fever and headache, to avoid delayed diagnosis.

## INTRODUCTION

In 2021, Maricopa County in Arizona faced its largest recorded West Nile virus outbreak, with 1,487 reported cases and 101 fatalities.^1^ Notably, neuroinvasive disease occurred in 956 of the identified cases.^1^ Emergency departments (ED) play a pivotal role in the early identification and management of infectious diseases like West Nile virus. However, variability in ED presentation can challenge recognition of the virus, especially when an outbreak takes place within the setting of the COVID-19 pandemic.

Most emergency physicians (EP) are aware of more common symptoms of neuroinvasive West Nile virus, which can present as meningitis, encephalitis, or acute paralysis. Patients with neurological involvement may present with fever, altered mental status, headaches, tremors, ataxia, bulbar dysfunction, stroke-like syndromes, myelitis, visual disturbance, and seizure activity.^2^ Gastrointestinal (GI) symptoms, including diarrhea, nausea and vomiting, also may predominate. However, presenting symptoms also include more vague complaints such as generalized weakness, malaise, chills, rash, fatigue, arthralgias, myalgias, and lymphadenopathy. Less commonly, patients may experience inflammatory processes of other organ sites, including ocular inflammation, myocarditis, hepatitis, and pancreatitis; rhabdomyolysis, stiff person syndrome, and autonomic instability have also been reported.^2^

Treatment for West Nile virus remains supportive, with ongoing trials investigating potential benefits from immunoglobulin and interferon. Despite the limited treatments available, prompt diagnosis remains essential, as delayed diagnosis may complicate public health efforts including targeted mosquito control, which could limit a spreading outbreak. Additionally, early diagnosis may decrease hospital resource use pursuing alternative diagnoses, providing more rapid answers to concerned patients and families.

To better understand characteristics of ED patients with West Nile virus, we conducted a retrospective chart review of encounters within the Mayo Clinic Arizona ED during the peak of the 2021 virus outbreak. We sought to describe clinical features and demographics of ED patients ultimately diagnosed with West Nile virus and frequency of presentation to the ED before their diagnosis. We examined in detail cases where EPs initially did not suspect the presence of the virus to assist with disease recognition during future outbreaks.

## METHODS

The Mayo Clinic Arizona ED is a tertiary-care facility in Phoenix, Arizona, which in 2021 served approximately 47,000 patients per year with 24 rooms and up to nine hallway spaces. All ED patients were treated by residency-trained EPs without nurse practitioner or physician assistant involvement. Approximately 5% of ED cases involved a rotating resident. All patients admitted to “observation status” and “inpatient status” were treated by inpatient hospitalists and, therefore, were considered “admitted” in our study. The EPs ordered West Nile virus diagnostics based on their clinical impression of a patient; if inpatient teams determined a patient needed a lumbar puncture after admission, radiology or neurology was engaged to perform the procedure.

Admission orders were placed to hospitalists after a phone discussion. Patients placed in progressive care or critical care units would move to their hospital bed only after evaluation by the accepting service, whereas patients admitted to floor wards would move upstairs after the phone conversation was complete. We manually reviewed charts to determine whether the EP or the hospitalist placed each West Nile virus test order. No specific education was provided to EPs during the virus outbreak regarding testing recommendations.

**Population Health Research Capsule**

What do we already know about this issue?

*Clinical presentations of West Nile virus can vary greatly, potentially complicating emergency department (ED) diagnosis*.

What was the research question?

*We sought to describe clinical features of ED patients ultimately diagnosed with West Nile virus and frequency of presentation before diagnosis*.

What was the major finding of the study?

*Most common chief complaints were fever (23.8%), headache (17.7%), and weakness (11.6%). Emergency physicians tested in 63/147 cases (42.9%). Those dispositioned without testing were older (median 73 vs 62 years; P < .001)*.

How does this improve population health?

*More comprehensive testing of the elderly with vague presentations may foster early diagnosis and public health interventions to limit spread*.

We performed a retrospective chart review to examine ED presentations of West Nile virus during the 2021 outbreak. We collected data from May–December 2021, coinciding with the peak of the outbreak.^1^ We extracted data from our hospital’s electronic health record, creating a database of all patients with a positive blood (serum antibody or whole blood polymerase chain reaction [PCR]) or cerebrospinal fluid (CSF) result for West Nile Virus and at least one visit to the ED within 15 days of the positive test. The incubation period for the virus is typically 2–14 days; thus, we selected 15 days to potentially catch early ED visits related to the start of illness. We excluded charts with only IgG-positive results, as these would have represented a past but not active infection. We also excluded charts with tests ordered outside our ED or inpatient settings, as pre-existing knowledge of a patient’s positivity for the virus may have affected ED presenting complaints or evaluation decisions. The final sample was comprised of 147 cases. One author (KJ) performed chart review to categorize patients, and a second author (NH) performed a secondary chart review to confirm correct categorization, with differences adjudicated by a third author (WM). We provide a PRISMA diagram displaying exclusions with counts as Appendix 1.

We extracted demographic information including age, sex, and race, clinical indicators including Emergency Severity Index (ESI) score, chief complaint, initial vital signs, and primary ED diagnosis, and diagnostic information including whether physicians performed blood or CSF tests for West Nile virus. Although most patients receiving viral blood testing underwent serum antibody testing, nine patients underwent whole blood PCR testing for West Nile virus; as the diagnostic process for both tests entailed a blood draw, we grouped these patients together as having received “blood” testing. We categorized patients who received both CSF and blood tests under CSF testing due to the more invasive nature of the lumbar puncture. Meningitis panels did not automatically include West Nile virus testing, and separate CSF order for the virus had to be placed.

All West Nile virus tests were shipped out-of-state for analysis. 3 The CSF and serum antibody tests were run at the receiving laboratory on Monday, Wednesday, and Friday, and whole blood PCR testing was performed daily Monday through Friday. Physicians were aware of the send-out nature of testing for West Nile virus and were not aware that whole blood PCR testing was run more frequently than serum antibody or CSF testing. Number of ED visits within 15 days of a positive West Nile virus test and ultimate disposition were recorded along with timing of the testing, including whether it was performed at the initial or subsequent ED visit. We evaluated whether patients were tested for WNV by the ED or whether the patient was admitted undifferentiated to an inpatient team. We compared the chief complaints between cases diagnosed by the ED and those not tested by an EP. A triage nurse categorized patient chief complaint via choices available in the electronic health record; options available to our nursing staff are provided in Appendix 2. Chief complaints of nausea and vomiting were combined to explore GI manifestations.

We summarized continuous variables using medians and interquartile ranges (IQR), while categorical variables were summarized using frequencies and percentages. We used the Kruskal-Wallis rank sum test for continuous variables and the Fisher exact test for categorical variables to compare groups of interest. A *P*-value of < .05 was considered statistically significant. We performed statistical analyses using R v4.1.2 (R Foundation for Statistical Computing, Vienna, Austria). All recommended methodological criteria described by Worster et al were followed other than abstractor blinding to hypothesis, which was not feasible as our first author served as abstractor. Chart review entailed recording of objective data from the health record; however, to limit potential bias, a secondary review was performed by an additional author to confirm agreement with patient categorization.^4^ The Mayo Clinic Arizona Institutional Review Board reviewed this study via expedited review procedures and deemed it exempt.

## RESULTS

We analyzed 147 West Nile virus ED presentations. Median patient age was 67.0 years of age (IQR 20.5) with male (66.7%) and White (97.3%) predominance, consistent with county-wide findings from the Maricopa County Department of Public Health’s post-epidemic report.^1^ The ESI breakdown was as follows: ESI 1, two cases (1.4%); ESI 2, 62 cases (42.2%); ESI 3, 81 cases (55.1%); ESI 4, two cases (1.4%); and ESI 5, 0 cases (0.0%).

We present EP testing decisions categorized by chief complaint in Table 1. Three patients, deemed unlikely to have initial presentations related to West Nile virus, were grouped together as “other” (chief complaints of urinary frequency, vascular access problem, and head injury).

The EPs tested patients during the first visit in 63 of 147 cases (42.9%). Twenty-two cases were tested and then discharged during their initial visit. The EPs admitted 104 cases during their initial visit; among these, they conducted West Nile virus testing in 41 cases. In patients discharged without testing on their first visit (21 cases), EPs performed the virus testing during a second ED visit in 17 cases and a third ED visit in four cases.

When comparing patients tested by an EP during the index visit to those who went untested by an EP, EPs initiated West Nile virus testing during the initial visit in younger patients (median [IQR] 62 [19] years tested vs 73 [14.2] years untested, *P* < .001), patients with higher oxygen saturation (97% [3] tested vs 96% [3] untested, *P* = .02), and lower respiratory rates (mean [SD] 18.3 (3.1) breaths/minute tested vs 19.4 (3.8) untested, *P* = .05). Patient sex, race, ESI, systolic blood pressure, temperature, and pulse rate were not associated with the EP’s decision to initiate testing during the initial ED visit.

The 21 patients discharged without West Nile virus testing were older (median [IQR] 77 [10] years of age discharged without testing vs 65.5 [21.8] tested or admitted, *P* = .002) and had lower temperatures (36.8 °C [0.6] not tested vs 37.2 °C [1.3] tested or admitted, *P* = .02) than those who received testing either inpatient or in the ED. The EPs discharged without testing patients with the following chief complaints: fever (three cases); “other” (three cases); abdominal pain (two cases), generalized weakness (two cases), and one case each of altered mental status, back pain, chest pain, chills, diarrhea, dizziness, fall, fatigue, flu symptoms, headache, and nausea/vomiting.

The EPs admitted but did not test patients with the following chief complaints: fever (15); generalized weakness (11); shortness of breath (8); altered mental status (5); nausea/vomiting (5); and headache (5). This category also included three cases each of falls and abdominal pain, and one case each of acute neurological problem, back pain, diarrhea, dizziness, fatigue, leg pain, stroke-like symptoms, and vertigo. We display EP-selected admission diagnoses for admitted patients who did not receive EP-ordered testing in Table 2, grouped intuitively for easy viewing. Of 63 patients who were admitted without EP-ordered West Nile virus testing, the inpatient team ordered the testing on 34 patients within 24 hours of patient arrival, with 49 patients receiving testing within 48 hours.

Forty-two patients diagnosed in the ED underwent CSF testing while 21 patients were diagnosed by serum alone. Age, sex, race, temperature, pulse rate, systolic blood pressure, respiratory rate, and oxygen saturation were not associated with the EP’s decision to test via CSF vs serum. Patients tested via CSF had lower ESI scores compared to serum (ESI score of 1–2, 45.3% vs 14.3%; *P* = .03).

## DISCUSSION

Of 147 West Nile virus cases, 21 patients required a return visit to the ED for diagnosis. Although some of these visits may have been for unrelated illnesses, the 15-day return visit timeframe suggests at least some of these may have represented a missed diagnosis at the index visit. Similarly, although our EPs recognized the severity of illness and admitted 104 initial visits, EPs only tested 41 of those patients for the virus. This suggests diagnostic difficulties related to broad initial ED West Nile virus presentations. Prior research supports this; in one study, only 5% of initially asymptomatic viremic West Nile virus blood donors were correctly diagnosed when ultimately seeking medical care.^2^,^5^

West Nile virus patients whom the EP declined to test tended to be older, with higher respiratory rates and lower oxygen saturations. Examining the cases that received no viral test during the initial ED visit, although many patients presented with typical meningitis or encephalitis complaints such as fever, headache, and altered mental status, many of the possible missed opportunities involved GI or respiratory issues, symptoms that have been shown to represent neuroinvasive disease.^2^,^6^,^7^ The EPs also commonly declined to test patients with vague complaints such as generalized weakness, falls, dizziness and fatigue; during other outbreaks, generalized weakness has been associated with ultimate diagnosis of encephalitis.^8^ In contrast, EPs did an excellent job testing the majority of West Nile virus headache chief complaints, which likely explains the CSF predominance of EP-initiated testing.

Examining Table 2, we find admission diagnoses that fit known West Nile virus presentations, such as stroke-like syndromes and rhabdomyolysis, along with more vague diagnoses such as generalized weakness. The diversity of possible presentations likely creates diagnostic difficulties for EPs functioning in a high-paced clinical environment. In five cases, EPs admitted a patient for presumed urinary tract infection who ultimately was diagnosed with West Nile virus; prior studies suggest that EPs may over-diagnose urinary tract infections in elderly patients.^9^ The EPs may have anchored on alternate pathology (such as a suggestive urinalysis) to explain weakness or confusion, especially in elderly patients and, therefore, may have not pursued testing for West Nile virus. Our EPs also declined to test 18 admitted patients with fever-related diagnoses for the virus. Our hospital serves many immunocompromised patients with transplants and cancer; thus, EPs may have anchored on bacteremia as the underlying cause of fever, leading to delayed diagnosis of West Nile virus.

Emergency physicians may benefit from directed educational interventions during known outbreaks to keep West Nile virus “top of mind” and on the differential, especially for vague complaints or rare West Nile virus presentations. Posted information discussing West Nile virus presentations or formal testing protocols could increase ED-based diagnosis, thereby decreasing the use of hospital resources to pursue alternative diagnoses and providing more rapid answers and early access to experimental treatments to patients and family.

## LIMITATIONS

Site EP and patient characteristics may have affected results. Our hospital cares for many elderly, immunocompromised patients; this may have altered patient presenting symptoms or staff testing patterns, as this population suffers increased risk of neuroinvasive disease.^2^,^10^ Alternately, as West Nile virus-infected immunocompetent patients may not be symptomatic, some West Nile virus-positive ED encounters may have had symptoms representing other concurrent illnesses.^8^ Additionally, our study did not include patients with positive West Nile virus tests in external hospitals or patients who tested positive outside the ED or in inpatient settings; there likely were patients who were discharged from our ED without initial West Nile virus testing and re-presented elsewhere and thus were not examined in our study. Although we attribute orders placed by an EP to his or her own clinical acumen, it is feasible that some tests were performed at the request of the inpatient team. Physician knowledge of the West Nile virus outbreak may have affected testing decisions, especially as the outbreak progressed. The EPs may also have viewed patient presentations differently in the setting of the concurrent COVID-19 pandemic.

## CONCLUSION

Emergency physicians did not initiate testing in 57.1% of initial ED encounters of patients ultimately found to have West Nile virus. During outbreaks of the virus, EPs should stay vigilant for less acute presentations, such as generalized weakness, GI complaints, and shortness of breath, particularly in elderly patients, alongside the more typical presentations of headache and fever. Emergency department leadership in areas prone to West Nile virus should consider creating educational materials or testing protocols to assist EPs, especially during active outbreaks.

## Supplementary Information





## Figures and Tables

**Table 1:** West Nile virus emergency department chief complaints during index visit.

No ED test (N = 84)	ED test (N = 63)
Fever (18)	Headache (20)
Weakness – generalized (13)	Fever (17)
Shortness of breath (8)	Altered mental status (6)
Altered mental status (6)	Nausea/vomiting (6)
Headache (6)	Weakness – generalized (4)
Nausea/vomiting (6)	Fatigue (3)
Abdominal pain (5)	Chest pain (2)
Fall (4)	Flu symptoms (1)
Other (3)	Generalized body aches (1)
Back pain (2)	Seizures (1)
Diarrhea (2)	Shortness of breath (1)
Dizziness (2)	Syncope (1)
Fatigue (2)	
Acute neurological problem* (1)	
Chest pain (1)	
Chills (1)	
Flu symptoms (1)	
Leg pain (1)	
Stroke-like symptoms (1)	
Vertigo (1)	

* Upon chart review, “acute neurological problem” meant bilateral lower extremity weakness.

*ED*, emergency department.

**Table 2:** Admission diagnoses for patients not receiving emergency department testing for West Nile virus.

Admitted, No ED test (N = 63)
Electrolyte-related diagnoses: diabetic ketoacidosis, hyponatremia
Fever-related diagnoses: fever (concern for COVID-19), fever of unknown origin ×12, fever with chill, sepsis ×3, SIRS
Gastrointestinal diagnoses: abdominal pain, diarrhea, intestinal obstruction, nausea and vomiting
Muscular diagnoses: back pain, myositis, rhabdomyolysis
Neurological diagnoses: aphasia, confusion, encephalopathy, headache, meningitis ×3, migraine, myelitis, stroke, vertigo
Respiratory diagnoses: pleural effusion, pneumonia ×3, shortness of breath ×4
Other: acute renal failure, alcohol withdrawal, generalized weakness ×8, hip fracture, palpitations, urinary tract infection ×5

*ED*, emergency department; *SIRS*, systemic inflammatory response syndrome.
