# Supplementary figures and images for: Emergency Department Presentations of West Nile Virus
Source: West J Emerg Med. 2025 Dec 24;27(1):214–8. doi: 10.5811/westjem.47475 (PMC12815563; doi:10.5811/westjem.47475)

**Appendix 1: PRISMA diagram for exclusions**


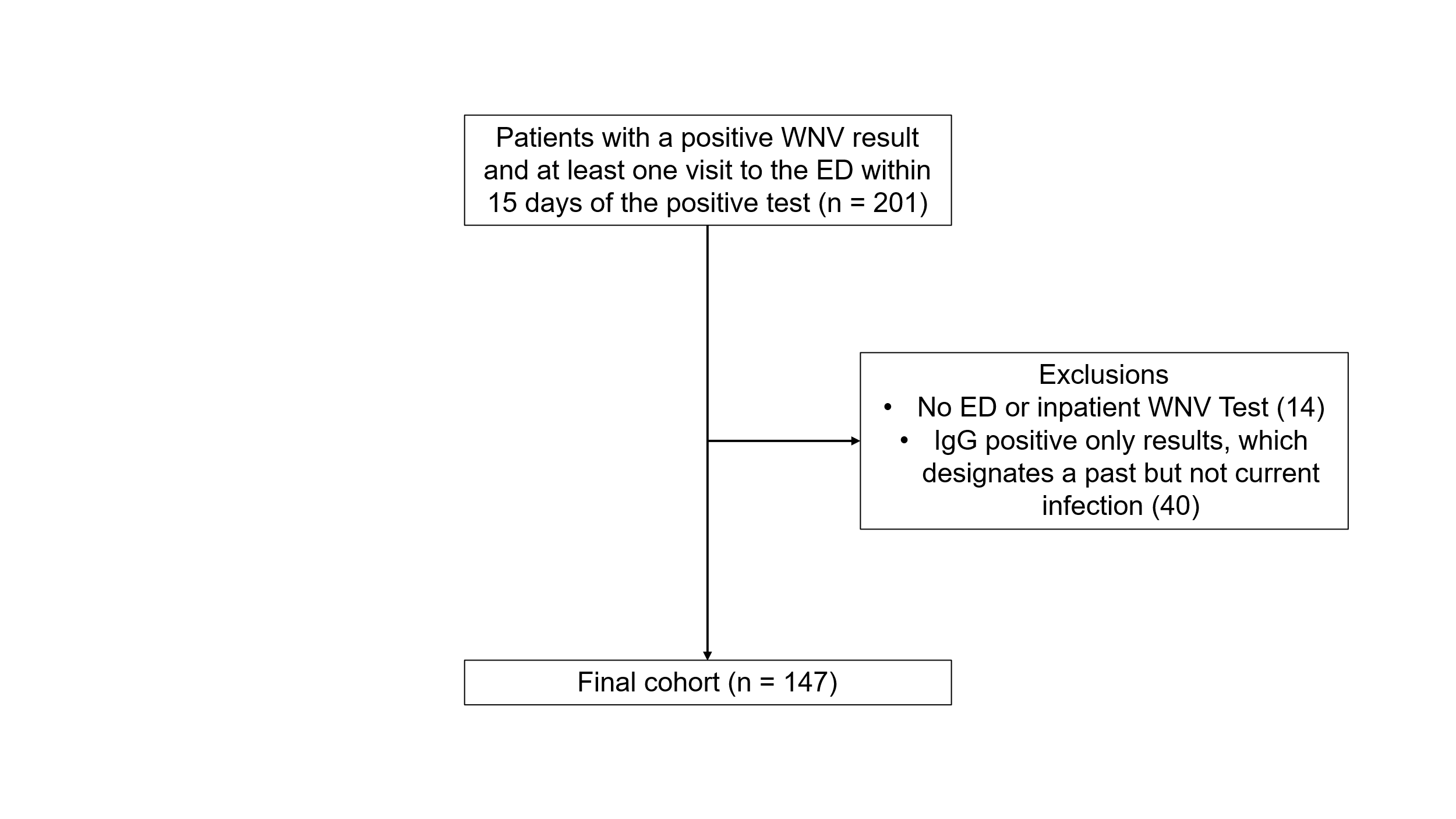

Supplement: Supplementary file 1 [file wjem-27-214-s001.docx]
