# Supplementary material for: Emergency Department Presentations of West Nile Virus
Source: West J Emerg Med. 2025 Dec 24;27(1):214–8. doi: 10.5811/westjem.47475 (PMC12815563; doi:10.5811/westjem.47475)
Supplement: Supplementary file 2 [file wjem-27-214-s002.docx]

**Appendix 2:** Electronic Medical Record chief complaint options

1 CHEST PAIN [100000]

2 SHORTNESS OF BREATH [100001]

3 DIZZINESS [100002]

4 PALPITATIONS [100004]

5 LEG SWELLING [100007]

6 STRESS [103]

7 SUTURE / STAPLE REMOVAL [105]

8 ABDOMINAL CRAMPING [110000]

9 ABDOMINAL INJURY [110001]

10 ABDOMINAL PAIN [110002]

11 RECTAL BLEEDING [110006]

12 RECTAL PAIN [110010]

13 DIFFICULTY SWALLOWING [110016]

14 ANEMIA [110029]

15 FOLLOW-UP [110033]

16 TRAUMA [112]

17 GASTROSTOMY TUBE CHANGE [1123]

18 URI [115]

19 NEWBORN DELIVERY [1161]

20 IMMINENT DELIVERY [1162]

21 BACK PAIN [12]

22 VOMITING [120]

23 LABORING [120012]

24 VAGINAL BLEEDING [120015]

25 BLINDNESS [130002]

26 EYE TRAUMA [130016]

27 EYE PAIN [130017]

28 FOREIGN BODY IN EYE [130021]

29 LOSS OF VISION [130027]

30 SPOTS AND/OR FLOATERS [130037]

31 HEAD INJURY [137]

32 ANKLE PAIN [140001]

33 ANKLE INJURY [140002]

34 ELBOW PAIN [140004]

35 ELBOW INJURY [140005]

36 FOOT PAIN [140006]

37 FOOT INJURY [140007]

38 HAND PAIN [140008]

39 HIP PAIN [140010]

40 KNEE PAIN [140012]

41 SHOULDER PAIN [140014]

42 WRIST PAIN [140016]

43 WRIST INJURY [140017]

44 CHRONIC RENAL FAILURE [1410000001]

45 ACUTE RENAL FAILURE [1410000012]

46 URINARY TRACT INFECTION [1410000014]

47 OUTPATIENT COVID-19 TESTING [1483]

48 CEREBROVASCULAR ACCIDENT [149]

49 LOSS OF SMELL [1490]

50 CHANGE IN TASTE SENSATION [1491]

51 FINGER INJURY [156]

52 BREAST PROBLEM [16]

53 HERNIA [160]

54 ABRASION [160007]

55 ADDICTION PROBLEM [160016]

56 AGGRESSIVE BEHAVIOR [160017]

57 AGITATION [160018]

58 AICD PROBLEM [160020]

59 AIRWAY OBSTRUCTION [160022]

60 ALCOHOL INTOXICATION [160023]

61 ASSAULT VICTIM [160026]

62 ALLEGED CHILD ABUSE [160027]

63 DOMESTIC VIOLENCE [160028]

64 SEXUAL ASSAULT [160029]

65 ALLERGIC REACTION [160030]

66 ALTERED MENTAL STATUS [160032]

67 UNABLE TO SPEAK [160045]

68 ARM INJURY [160050]

69 ARM SWELLING [160052]

70 ASPIRATION [160053]

71 BLISTER [160070]

72 BLOATED [160071]

73 BLOOD INFECTION [160074]

74 BODY FLUID EXPOSURE [160077]

75 BREAST MASS [160078]

76 BREAST PAIN [160079]

77 CARDIAC ARREST [160088]

78 CAST PROBLEM [160089]

79 CAST REPAIR [160091]

80 CHEMICAL EXPOSURE [160094]

81 CHEST INJURY [160096]

82 CHILLS [160101]

83 CHOKING [160102]

84 CIRCULATORY PROBLEM [160103]

85 COLLARBONE INJURY [160104]

86 COAGULATION DISORDER [160109]

87 COLD EXPOSURE [160110]

88 COLD EXTREMITY [160111]

89 NASAL CONGESTION [160114]

90 CYST [160122]

91 DEAD ON ARRIVAL [160123]

92 SKIN ULCER [160124]

93 DEHYDRATION [160126]

94 DELIRIUM TREMENS (DTS) [160127]

95 DENTAL INJURY [160128]

96 DENTAL PAIN [160129]

97 ORAL SWELLING [160131]

98 DIAPER RASH [160133]

99 DISLOCATION [160138]

100 DRAINAGE FROM INCISION [160143]

101 DRESSING CHANGE [160144]

102 DROOLING [160146]

103 ADDICTION ASSESSMENT [160147]

104 EAR DRAINAGE [160151]

105 EAR INJURY [160152]

106 EAR LACERATION [160153]

107 EARACHE [160154]

108 EATING DISORDER [160157]

109 ELECTRIC SHOCK [160169]

110 NOSE BLEED [160173]

111 ERECTILE DYSFUNCTION [160174]

112 EXPOSURE TO STD [160178]

113 EXTREMITY WEAKNESS [160179]

114 EYE DRAINAGE [160180]

115 FACIAL BURN [160189]

116 FACIAL DROOP [160190]

117 FACIAL INJURY [160191]

118 FACIAL LACERATION [160192]

119 FACIAL PAIN [160194]

120 FACIAL SWELLING [160196]

121 FALL [160198]

122 HANGING [160200]

123 NECK STRANGULATION [160201]

124 FLANK PAIN [160219]

125 FOOT BURN [160223]

126 MENTAL HEALTH PROBLEM [160224]

127 FOREIGN BODY IN EAR [160232]

128 FOREIGN BODY IN NOSE [160234]

129 FOREIGN BODY IN RECTUM [160235]

130 FOREIGN BODY IN VAGINA [160237]

131 SWALLOWED FOREIGN BODY [160239]

132 FROSTBITE [160240]

133 FUSSY [160241]

134 GAIT PROBLEM [160243]

135 GENERALIZED BODY ACHES [160245]

136 GI PROBLEM [160246]

137 SWOLLEN GLANDS [160247]

138 GROIN INJURY [160248]

139 GROIN PAIN [160250]

140 GROIN SWELLING [160252]

141 GINGIVITIS [160253]

142 GUN SHOT WOUND [160254]

143 HALLUCINATIONS [160255]

144 HAND BURN [160256]

145 HAND INJURY [160258]

146 HEAD LACERATION [160267]

147 HEAD LICE [160268]

148 HEAT EXPOSURE [160277]

149 VOMITING BLOOD [160278]

150 BLOOD IN URINE [160279]

151 COUGING UP BLOOD [160280]

152 HEMORRHOIDS [160281]

153 HICCUPS [160283]

154 HYPERGLYCEMIA [160285]

155 HOARSE [160296]

156 HOMELESS [160298]

157 HOMICIDAL [160299]

158 HUMAN BITE [160300]

159 HYPERTENSION [160302]

160 HYPERVENTILATING [160304]

161 INCISIONAL PAIN [160308]

162 TOXIC INHALATION [160312]

163 INJURY [160313]

164 INSECT BITE [160314]

165 INSOMNIA [160315]

166 INSULIN REACTION [160316]

167 INGESTION [160317]

168 IRREGULAR HEART BEAT [160319]

169 IV MEDICATION [160323]

170 JAUNDICE [160325]

171 JAW PAIN [160329]

172 JOINT SWELLING [160337]

173 KNEE INJURY [160340]

174 LABS ONLY [160350]

175 LEG INJURY [160355]

176 LEG PAIN [160357]

177 LETTER FOR SCHOOL/WORK [160363]

178 LIGHTNING STRIKE [160365]

179 LIP LACERATION [160367]

180 HYPOGLYCEMIA [160376]

181 MANIC BEHAVIOR [160380]

182 MEDICATION REFILL [160383]

183 MEDICATION REACTION [160384]

184 BLACK OR BLOODY STOOL [160386]

185 MISCARRIAGE [160390]

186 AMENORRHEA [160391]

187 EMERGENCY CONTRACEPTION [160392]

188 MORNING SICKNESS [160394]

189 MOTOR VEHICLE CRASH [160395]

190 MOTORCYCLE CRASH [160396]

191 MOTORCYCLE VS PEDESTRIAN [160397]

192 MOUTH INJURY [160398]

193 MOUTH LESIONS [160400]

194 MUSCLE PAIN [160406]

195 NASAL POLYPS [160414]

196 NEAR DROWNING [160418]

197 NECK INJURY [160421]

198 NECK PAIN [160423]

199 CONSTIPATION [160431]

200 DRUG OVERDOSE [160436]

201 PACEMAKER PROBLEM [160439]

202 PANIC ATTACK [160443]

203 PARALYSIS [160445]

204 PENILE DISCHARGE [160450]

205 PERSONAL PROBLEM [160457]

206 PAIN WITH BREATHING [160459]

207 POISON IVY [160460]

208 POISONING [160461]

209 POSTPARTUM COMPLICATIONS [160467]

210 POST-OP PROBLEM [160471]

211 TB TEST [160472]

212 POSSIBLE PREGNANCY [160474]

213 ITCHING [160476]

214 PUNCTURE WOUND [160480]

215 RAPID HEART RATE [160481]

216 RASH [160482]

217 FOREIGN BODY IN SKIN [160486]

218 RESPIRATORY ARREST [160489]

219 RESPIRATORY DISTRESS [160490]

220 RIB INJURY [160492]

221 TINNITUS [160496]

222 RUNAWAY [160497]

223 SHAKING [160503]

224 SHOULDER INJURY [160507]

225 SICKLE CELL PAIN CRISIS [160514]

226 BRADYCARDIA [160526]

227 SMOKE INHALATION [160528]

228 SNAKE BITE [160529]

229 SNORING [160530]

230 SORE [160533]

231 SPASMS [160536]

232 STAB WOUND [160541]

233 TORTICOLLIS [160548]

234 EAR FULLNESS [160549]

235 SUICIDAL [160555]

236 SUICIDE ATTEMPT [160556]

237 SUNBURN [160558]

238 NIGHT SWEATS [160561]

239 TAILBONE PAIN [160564]

240 TEETHING [160566]

241 THREATENED MISCARRIAGE [160574]

242 TICK REMOVAL [160576]

243 TINGLING [160577]

244 TEMPOROMANDIBULAR JOINT PAIN [160578]

245 TOE INJURY [160580]

246 TOE PAIN [160582]

247 TRACHEOSTOMY TUBE CHANGE [160589]

248 TREMORS [160594]

249 URINARY FREQUENCY [160605]

250 DIFFICULTY URINATING [160606]

251 URINARY RETENTION [160607]

252 VAGINAL PROLAPSE [160608]

253 VAGINAL DISCHARGE [160611]

254 VAGINAL ITCHING [160613]

255 VASCULAR ACCESS PROBLEM [160618]

256 VOMITING DURING PREGNANCY [160623]

257 CERUMEN IMPACTION [160626]

258 WEIGHT LOSS [160628]

259 WHEEZING [160630]

260 WITHDRAWAL [160632]

261 WOUND CHECK [160636]

262 WOUND DEHISCENCE [160637]

263 WOUND INFECTION [160638]

264 CYSTITIS [160652]

265 VARICELLA [160659]

266 CONCUSSION [160664]

267 CROUP [160671]

268 GERD [160679]

269 FEBRILE SEIZURE [160680]

270 FECAL IMPACTION [160681]

271 INFLUENZA [160682]

272 GESTATIONAL DIABETES [160688]

273 HEARTBURN [160690]

274 MIGRAINE [160698]

275 PANCREATITIS [160700]

276 PRE-ECLAMPSIA [160704]

277 SINUSITIS [160713]

278 TRANSIENT ISCHEMIC ATTACK [160720]

279 PARANOIA [160722]

280 DELUSIONAL [160723]

281 VAGINITIS [160725]

282 BREAST DISCHARGE [160731]

283 PSYCHIATRIC EVALUATION [160737]

284 WEAKNESS - GENERALIZED [160786]

285 VAGINAL BLEEDING - PREGNANT [160787]

286 STROKE [160788]

287 LOWER EXTREMITY ISSUE [160789]

288 UPPER EXTREMITY ISSUE [160790]

289 DIFFICULTY BREATHING [160900]

290 PREGNANCY PROBLEM [160999]

291 WEIGHT GAIN [180]

292 CAST REMOVAL [20]

293 JOINT PAIN [200100]

294 ABNORMAL LAB [200101]

295 HERPES ZOSTER [206]

296 INGUINAL HERNIA [249]

297 FOREIGN BODY [264]

298 ILLNESS [265]

299 MALE GU PROBLEM [266]

300 ABSCESS [267]

301 SKIN PROBLEM [270]

302 COUGH [28]

303 ALCOHOL PROBLEM [3]

304 DEMENTIA [30]

305 DENTAL PROBLEM [31]

306 DEPRESSION [32]

307 DEVICE CHECK [3371]

308 URINARY URGENCY [343]

309 SCIATICA [346]

310 DIARRHEA [35]

311 HYPOTENSION [407]

312 FINGER LACERATION [412]

313 EYE PROBLEM [43]

314 FATIGUE [46]

315 FEVER [47]

316 ACUTE INTOXICATION [504]

317 FLU SYMPTOMS [505]

318 SYNCOPE [506]

319 HEADACHE [52]

320 HEARING PROBLEM [53]

321 HIVES [56]

322 URINARY PROBLEM [563]

323 NURSE VISIT [565]

324 VERTIGO [576]

325 ABNORMAL POTASSIUM [586]

326 ABNORMAL SODIUM [590]

327 MEDICATION VISIT [612]

328 MASS [64]

329 MEMORY LOSS [66]

330 NAUSEA [70]

331 NUMBNESS [75]

332 SORE THROAT [82]

333 BURN [84]

334 LACERATION [86]

335 ANIMAL BITE [87]

336 RED EYE [879]

337 EYE SWELLING [881]

338 ABDOMINAL DISTENTION [882]

339 NASAL TRAUMA [884]

340 ELEVATED BP [885]

341 MYALGIA [886]

342 BICYCLE ACCIDENT [887]

343 PEDESTRIAN TRAUMA [888]

344 HEART PAIN [889]

345 PERITONSILLAR ABSCESS [890]

346 SCABIES [891]

347 BEDBUGS [892]

348 NEAR SYNCOPE [893]

349 ANAPHYLAXIS [894]

350 ANGINA [895]

351 PAINFUL URINATION [896]

352 UNRESPONSIVE [897]

353 PAROXYSMAL POSITIONAL VERTIGO [898]

354 CONGESTION [899]

355 ANXIETY [9]

356 FOCAL WEAKNESS [900]

357 PARESTHESIA [901]

358 MINI STROKE [902]

359 AUTO VERSUS PEDESTRIAN [903]

360 DIABETIC WOUND [904]

361 NON HEALING WOUND [905]

362 PRESSURE ULCER [906]

363 PERIANAL ITCHING [907]

364 PERINEAL LESION [908]

365 RECTAL DISCHARGE [909]

366 HOMICIDAL IDEATION [910]

367 MONO [911]

368 NEUTROPENIA [912]

369 INCREASED WORK OF BREATHING [913]

370 BEHAVIORAL DYSCONTROL [914]

371 PARENT CHILD DISCORD [915]

372 HYPHEMA [916]

373 DEVICE MALFUNCTION [934]

374 DYSURIA [935]

375 INFLUENZA LIKE ILLNESS [938]

376 DIABETIC ULCER [939]

377 STROKE LIKE SYMPTOMS [940]

378 VOICE CHANGES [941]

379 STOMACH FLU [942]

380 SEIZURES [97]

381 BABY WEIGHT [982]

382 CHEMO PUMP DISCONTINUATION [983]

383 BLOOD PRODUCT ADMINISTRATION [984]

384 IM MEDICATION (ADMINISTRATION) [985]

385 URINARY CATHETER CHANGE [986]

386 SLURRED SPEECH [987]

387 ARM DRIFT [988]

388 INAPPROPRIATE WORDS [989]

389 ORTHOPNEA [990]

390 PAROXYSMAL NOCTURNAL DYSPNEA [991]

391 DIAPHORETIC [992]

392 ECG CHANGES [993]

393 EYE EXPOSURE [994]

394 AUDITORY HALLUCINATIONS [995]

395 STREP EXPOSURE [996]

396 HUMERUS INJURY [997]

397 FOREARM INJURY [998]

398 FEMUR INJURY [999]

399 HEART ASSIST DEVICE [812]

400 LUNG TRANSPLANT EVALUATION [2050000002]

401 KIDNEY TRANSPLANT EVALUATION [2050000011]

402 LIVER TRANSPLANT EVALUATION [2050000008]

403 INSULIN PUMP VISIT [788]

404 KIDNEY TRANSPLANT FOLLOW-UP [2050000012]

405 LUNG TRANSPLANT FOLLOW-UP [2050000003]

406 TRANSPLANT FOLLOW-UP [829]

407 ARM PAIN [160049]

408 CHEST WALL PAIN [1444]

409 CELLULITIS [315]

410 TUBE PROBLEM [1445]

411 NEUROLOGIC PROBLEM [71]

412 EXTREMITY LACERATION [152]

413 ATRIAL FIBRILLATION [80]

414 PELVIC PAIN [174]

415 COVID INQUIRY [1485]

416 FOOD BOLUS [875]

417 ASTHMA [148]

418 ABNORMAL TEST RESULT [1343]

419 BLURRED VISION [130003]

420 GI BLEEDING [238]

421 EAR PROBLEM [196]

422 BREATHING PROBLEM [17]

423 THROAT PROBLEM [567]

424 TESTICLE PAIN [260]

425 BLEEDING/BRUISING [14]

426 VAGINAL PAIN [259]

427 FOOT SWELLING [160231]

428 PNEUMONIA [164]

429 LEG PROBLEM [232]

430 FINGER PAIN [545]

431 HIP INJURY [140011]

432 PAIN [136]

433 SWELLING [762]

434 FOOT PROBLEM [229]

435 BACK INJURY [155]

436 ABNORMAL ECG [293]

437 ABNORMAL OXIMETRY [734]

438 COVID-19 REMOTE PATIENT MONITORING [1487]

439 FAILURE TO THRIVE [147]

440 POOR APPETITE [711]

441 THROMBOPHILIA [549]

442 DIPLOPIA [130011]

443 NEUTROPENIC FEVER [957]

444 OSTOMY CARE [613]

445 PENIS PAIN [258]

446 SINUS PROBLEM [99]

447 HAND PROBLEM [231]

448 DECREASED VISUAL ACUITY [130034]

449 IMMUNIZATIONS [58]

450 LOSS OF APPETITE [977]

451 ARM PROBLEM [226]

452 UROLITHIASIS [539]

453 RESPIRATORY ILLNESS [1321]

454 ASCITES [295]

455 ANGIOEDEMA [90]

456 CONJUNCTIVITIS [130032]

457 FOOT INFECTION [637]

458 ORAL PAIN [138]

459 OXYGEN DEPENDENCE [1228]

460 NOSE PROBLEM [262]

461 NAIL PROBLEM [1200000011]

462 TOE PROBLEM [627]

463 DEEP VEIN THROMBOSIS [456]

464 NEPHROLITHIASIS [160695]

465 DIABETIC KETOACIDOSIS [272]

466 BEHAVIOR PROBLEM [298]

467 DIFFICULTY WALKING [1040000003]

468 DIVERTICULITIS [132]

469 LEG CRAMPS [1603571]

470 BRONCHITIS [310]

471 CHRONIC PAIN [769]

472 MEDICATION PROBLEM [65]

473 BLOOD SUGAR PROBLEM [160075]

474 PENIS INJURY [256]

475 ABNORMAL X-RAY [564]

476 ACUTE NEUROLOGICAL PROBLEM [520]

477 KNEE PROBLEM [222]

478 PYELONEPHRITIS [1410000009]

479 RESTLESS LEG SYNDROME [398]

480 CONTRACEPTION [60]

481 INGROWN TOENAIL [214]

482 PRIAPISM [1404]

483 TICK EXPOSURE [1336]

484 AV FISTULA [2101870001]

485 BEHAVIORAL PROBLEM [1152]

486 CONTRACTIONS [100563]

487 HEMATOCHEZIA [253]

488 HIP PROBLEM [221]

489 UMBILICAL HERNIA [1125]

490 VISUAL FIELD CHANGE [130023]

491 STI SCREENING [552]

492 ACHILLES PAIN [626]

493 ALLERGIES [255]

494 DYSPHASIA [1459]

495 STRIDOR [1226]

496 TOENAIL PROBLEM [639]

497 ATRIAL FLUTTER [101]

498 FECAL INCONTINENCE [1458]

499 CYANOSIS [123]

500 BLOOD PRESSURE CHECK [299]

501 DIAPHORESIS [12000024]

502 MEMORY COMPLAINTS [1203]

503 MOVEMENT DISORDER [1207]

504 APNEA [100]

505 SLEEPINESS [735]

506 PHOTOPHOBIA [130026]

507 PLEURAL EFFUSION [1019]

508 POLYDIPSIA [190]

509 SPINE FRACTURE [979]

510 TESTICLE INJURY [261]

511 VAGINAL INJURY [257]

512 AMPUTATION [1076]

513 AVULSION [1087]

514 DETACHED RETINA [130010]

515 FLASHES, LIGHT [130044]

516 LARYNGITIS [2090000003]

517 NEUTROPENIA [333]

518 OUTPATIENT INFUSION [1150001]

519 POLYURIA [191]

520 PRETERM PREMATURE RUPTURE OF MEMBRANES [543]

521 TESTING FOR UPPER RESPIRATORY VIRUS SYMPTOMS [1495]

522 LOSS OF CONSCIOUSNESS [160372]

523 THRUSH [205]

524 INFECTION [546]

525 PULMONARY EMBOLISM [1021]

526 WOUND CARE [617]

527 DRUG PROBLEM [37]

528 FEEDING TUBE [608]

529 DRUG SCREEN [1324]

530 SPEECH PROBLEM [271]

531 RECTAL PROLAPSE [406]

532 FEEDING PROBLEM [475]

533 MENORRHAGIA [169]

534 DRUG/ALCOHOL DEPENDENCY [408]

535 MENSTRUAL PROBLEM [67]

536 NEUROPATHY [1039]

537 CROHN'S DISEASE [233]

538 HOT FLASHES [326]

539 DYSARTHRIA [478]

540 FEMALE GENITAL ISSUE [521]

541 STOOL COLOR CHANGE [110024]

542 CHEST WALL MASS [745]

543 RHINITIS [1097]

544 FEMALE GU PROBLEM [263]

545 SKIN REDNESS [1540]

546 CHIN LACERATION [1541]
